# A Novel Deep Supervised Learning-Based Approach for Intrusion Detection in IoT Systems

**DOI:** 10.3390/s22124459

**Published:** 2022-06-13

**Authors:** Sahba Baniasadi, Omid Rostami, Diego Martín, Mehrdad Kaveh

**Affiliations:** 1 Department of Industrial Engineering, University of Houston, Houston, TX 77204, USA; sbaniasa@cougarnet.uh.edu (S.B.); orostami@cougarnet.uh.edu (O.R.); 2 ETSI Telecomunicación, Universidad Politécnica de Madrid, 28040 Madrid, Spain; m.kaveh11@email.kntu.ac.ir

**Keywords:** IoT, network intrusion detection, deep learning, optimal network training

## Abstract

The Internet of Things (IoT) has become one of the most important concepts in various aspects of our modern life in recent years. However, the most critical challenge for the world-wide use of the IoT is to address its security issues. One of the most important tasks to address the security challenges in the IoT is to detect intrusion in the network. Although the machine/deep learning-based solutions have been repeatedly used to detect network intrusion through recent years, there is still considerable potential to improve the accuracy and performance of the classifier (intrusion detector). In this paper, we develop a novel training algorithm to better tune the parameters of the used deep architecture. To specifically do so, we first introduce a novel neighborhood search-based particle swarm optimization (NSBPSO) algorithm to improve the exploitation/exploration of the PSO algorithm. Next, we use the advantage of NSBPSO to optimally train the deep architecture as our network intrusion detector in order to obtain better accuracy and performance. For evaluating the performance of the proposed classifier, we use two network intrusion detection datasets named UNSW-NB15 and Bot-IoT to rate the accuracy and performance of the proposed classifier.

## 1. Introduction

The Internet of Things (IoT), as a new communication/control platform, connects everything/everybody to the Internet, where the behavior of connected nodes in the IoT can be monitored to or their operation can be controlled by a (group of) server(s) [1,2,3]. IoT-connected nodes can range from simple sensors in various environments to critical components in different applications and communicate with each other on a predefined (Internet-based) network [4]. With the global activation of the IoT, there will be a fundamental change in various aspects of human life, including industry, culture, education, trade, transportation, etc. Therefore, the IoT is one of the most important technologies being developed in the world today [5,6,7,8].

In general, there are several challenges facing the IoT in order for it to be practically implemented and move from the research and development to the productivity stage [9,10,11,12,13]. These challenges include but are not limited to: (a) large-scale: in any communication networks, there are different parameters that may lead to a decrease in the network performance, e.g., scalability, device heterogeneity, variety of network interactions, and network mobility rate [14]; (b) lack of infrastructure: in the IoT, the connected devices need to discover each other through a certain infrastructure [15]; and (c) commercialization: the International Telecommunication Union (ITU) has described the IoT-commercialization process as an important challenge and reported it as follows: “Many centers such as standard development organizations, research centers, service providers, network operators need to work together and each change many of its own rules and regulations” [16].

However, the most important and critical challenge that the IoT is facing, and will always face, is security. Security itself can be defined/considered in different aspects such as the kind of security requirements and threat models, the studied layer in the network, and the type of cryptographic primitives that can be used [17,18,19,20,21,22,23,24,25]. The same as most consumer technologies, IoT has not been considered with security in mind in the first place, leading security to be emerging as an important obstacle in the adoption of different networks and services.

**Figure 1:**
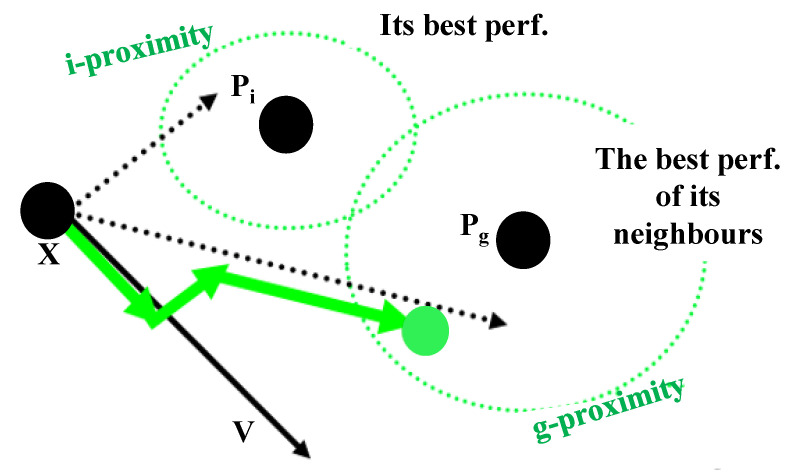
The velocity motions of particles in PSO.

Among all security mechanisms, intrusion detection [26,27,28] is one of the most important security mechanisms, which can be studied in all of four IoT architecture layers as depicted in Figure 1 [29]. The network intrusion detection system (NIDS) is known as a promising solution to detect the intrusion of malicious behaviors in IoT networks. The NIDS is mainly provided by the network layer in the IoT, which plays as a spine in order to connect various IoT devices. The adversarial threats in the network layer can be classified in four main categories including probing, denial of service (DoS), user to root (U2R), and remote to local (R2L) [30,31,32,33,34,35,36]. Another categorization for the NIDS is based on the scheme’s ability in detecting the intrusion, based on which it is divided into two main categories named signature-based intrusion detection and anomaly-based intrusion detection [37,38,39]. A more general classification can include the host intrusion detection (HID) and network intrusion detection (NID) [40,41,42], which have their own advantages and drawbacks.

### 1.1. Rekated Works

Through recent years, many schemes have been introduced for NID to better classify different attacks/threats in the network’s normal traffic. The traditional detection schemes have often employed statistical approaches, for example, distance measuring [43], the Hidden Markov Model (HMM) [44], Bayes theory [45], cluster analysis [46], and signal processing [47]; however, these methods have gradually given way to machine learning-based approaches. Thaseen et al. [48] introduced an approach using the support vector machine (SVM) and principal component analysis (PCA). They could improve the accuracy and training-time cost for some attacks in the network, e.g., U2R and R2L, by automatically tuning the optimization parameters and optimizing SVM’s kernels and parameters.

There are other well-known machine learning-based methods for detecting the attacks in IoT networks, including the multi-layer perceptron neural network (MLP NN), Random Forest (RF), and Naive Bayes (NB) [49,50,51,52,53], though, it has been shown that the performances of MLP, RF, NB, and other traditional machine learning-based approaches are not sufficient, especially when the number of traffic data is big, mostly because of their shallow learning essence. As a result of the growth in using deep learning in various ranges of applications, many efforts have been also done to propose an efficient and accurate NIDS based on deep learning. 

Yin et al. [54] have introduced a NIDS using a recurrent neural network (RNN). In comparison with former machine learning-based approaches, their scheme could obtain better classification accuracy and a higher detection rate. He et al. [55] have introduced a NIDS using the long short-term memory (LSTM) and multimodal deep auto-encoder for obtaining better accuracy. Garg et al. [56] have introduced an IoT NIDS based on the grey wolf optimizer (GWO) and the deep convolutional neural network (DCNN). The authors in [56] have shown that their proposed model could achieve a higher detection rate with minimized features on three network intrusion datasets. Xu et al. [57] proposed employing a log-cosh conditional variational auto-encoder (CVAE) in order to catch the complicated propagation of the observed data and produce new data with pre-specified classes, leading to the creation of a more efficient way to produce various intrusion data for disbalanced classes.

Deep learning-based approaches could have improved the accuracy of the NIDS, though there were still some important features that needed to be improved, including achieving a higher detection rate and decreasing the computational cost. One important thing to do on these scores, which has been rarely considered in the literature, is to optimally train the fully connected neural network in the deep architecture [58,59,60,61,62,63]. Due to the fact that better training the fully connected neural network leads to better classification accuracy, the used classifier can be designed in a more lightweight manner (in an equal detection rate), and thus less data will be required to train the network.

### 1.2. Paper Contributions

According to the drawbacks of the mentioned NID models, the most important contributions of this paper are summarized as follows:

- We improve a novel meta-heuristic algorithm named NSBPSO, in which new concepts such as employed bees, onlooker bees, and the multi-parent crossover of bees are introduced to better the exploitation and exploration abilities of the PSO algorithm.
- We optimally improve the performance of the DCNN as our NIDS by updating its optimization parameters using the NSBPSO algorithm.
- We evaluate the performance of the proposed evolutionary deep learning-based IDS by comparing it with other IoT intrusion detectors in the literature using the UNSW-NB15 [64] and Bot-IoT [65] datasets.

### 1.3. Paper Organization

The rest of this paper is organized as follows: Section 2 elaborates the proposed NSBPSO algorithm. Section 3 explains the proposed NIDS for the IoT, including the used datasets and the way of training the intrusion detector (DCNN) by the proposed NSBPSO algorithm. Section 4 evaluates the performance of the proposed evolutionary deep learning-based IDS by comparing it with other IoT intrusion detectors in the literature using the UNSW-NB15 [64] and Bot-IoT [65] datasets, and, finally, we conclude the paper in Section 5.

## 2. The Proposed NSBPSO Algorithm

Particle Swarm Optimization (PSO) is one of the most important meta-heuristic algorithms that was introduced by Kennedy and Eberhart in 1995. This algorithm was inspired by the social behavior of animals such as fish and birds. PSO is suitable for discrete and continuous problems and has performed very well in various engineering optimization problems.

**Figure 2:**
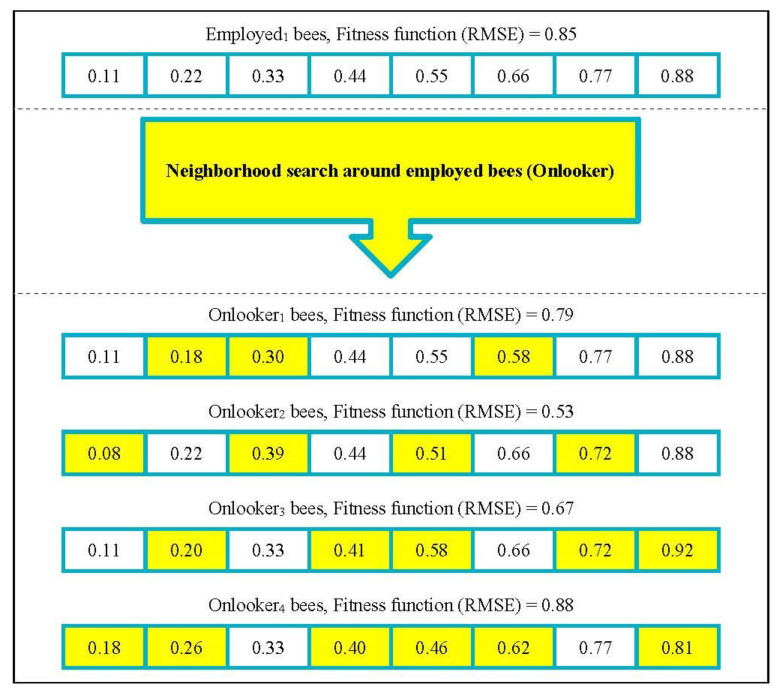
The example of a neighborhood search around employed bees.

In the PSO algorithm, solutions are mapped to particles, and each particle is assigned an initial velocity. The fitness function is used to calculate the next velocity of the particles in the search space. Particle velocity consists of three main movements: (a) the percentage of the previous movement, (b) the motion toward the best personal experience, and (c) the motion toward the best experience of other particles. Figure 2 indicates an overview of particle velocity motions in the PSO algorithm. Equations (1) and (2) represent the velocity and position of the particles, respectively.

(1) $$V_{id}\left(t+1\right)=\alpha V_{id}\left(t\right)+\beta \mathrm{rand}\left(0,\varphi_{1}\right)\left(P_{id}\left(t\right)-X_{id}\left(t\right)\right)+\beta \mathrm{rand}\left(0,\varphi_{2}\right)\left(P_{gd}\left(t\right)-X_{id}\left(t\right)\right)$$

(2) $$X_{id}\left(t+1\right)=X_{id}\left(t\right)+V_{id}\left(t+1\right)$$

where $V_{id}\left(t\right)$ = the current velocity of particle in dimension, d, $V_{id}\left(t+1\right)\;$= the new velocity of particle in dimension, d, $X_{id}\left(t\right)\;$= the current position of particle in dimension, d, $X_{id}\left(t+1\right)\;$= the new position of particle in dimension d; $\beta \mathrm{rand}\left(0,\varphi_{1}\right)\;$ = a random number between zero and $\varphi_{1}$, $\beta \mathrm{rand}\left(0,\varphi_{2}\right)\;$ = a random number between zero and $\varphi_{2}$, α = the inertial coefficient, $P_{id}\left(t\right)\;$ = the best personal experience of particles in dimension d, and $P_{gd}\left(t\right)\;$ = the best global experience of particles in dimension d.

This paper shows that standard PSO has two main drawbacks: (I) insufficient ability to explore and exploit solutions, and (II) getting stuck in local minimums. PSO has no operator to make sudden changes, which leads to getting stuck in local minimums. The PSO algorithm improves its position by considering the best personal and global experience. If the initial populations are far from the best solution, PSO can rarely converge. Another weakness of PSO is that this algorithm is highly dependent on the distribution of initial particles in the search space. If a considerable number of particles are trapped in local minimums, PSO can slightly prevent particles from being trapped in local minimums. However, PSO converges faster if the particles change suddenly. In this paper, to improve the PSO algorithm, employed bees, onlooker bees, and the multi-parent crossover of bees are used to amplify exploitation and exploration. The proposed algorithm is called neighborhood search-based particle swarm optimization (NSBPSO).

**Figure 3:**
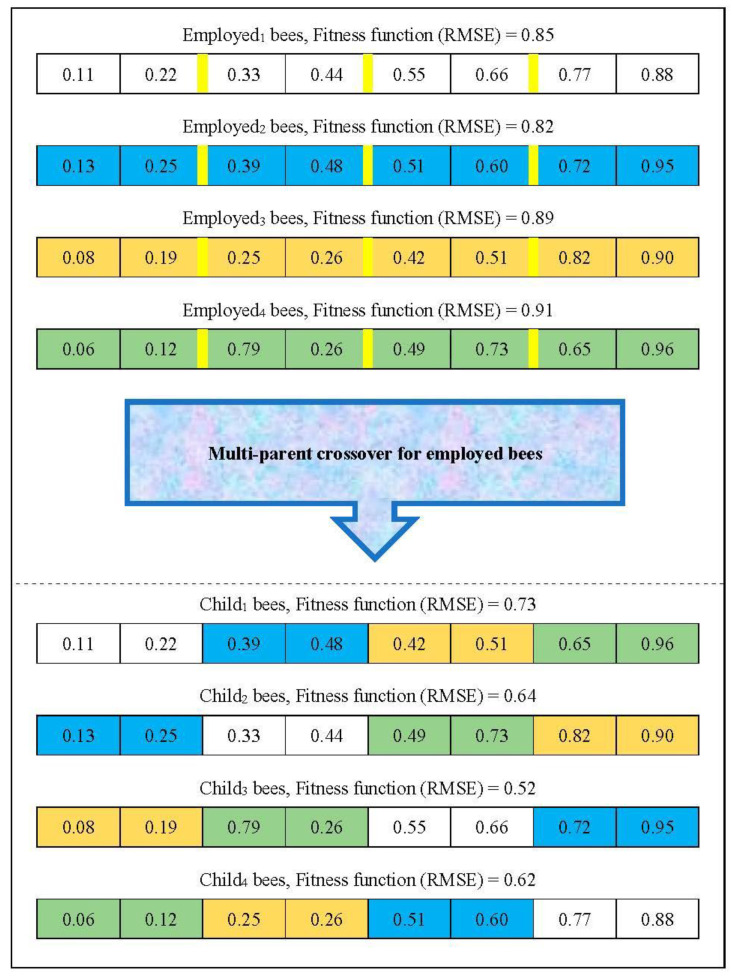
The example of the multi-parent crossover operator.

In the proposed NSBPSO algorithm, by considering several particles as the employed bees (global bests), different parts of the search space can be examined simultaneously. Therefore, it helps the algorithm to avoid being trapped in the local minimums. In the artificial bee colony (ABC) algorithm, the onlooker bees are obtained by a neighborhood search around the employed bees. If the onlooker bees are more efficient than the employed bees, they will be replaced by the employed bees and the employed bees will be updated. In the proposed NSBPSO algorithm, after selecting the employed bees, a number of onlooker bees are sent to search around them. Updated employed bees are then compared to the global best, and the global best is updated. In NSBPSO, onlooker bees play the role of exploiting good solutions. Figure 3 shows the example of the production of onlooker bees (a neighborhood search around employed bees).

**Figure 4:**
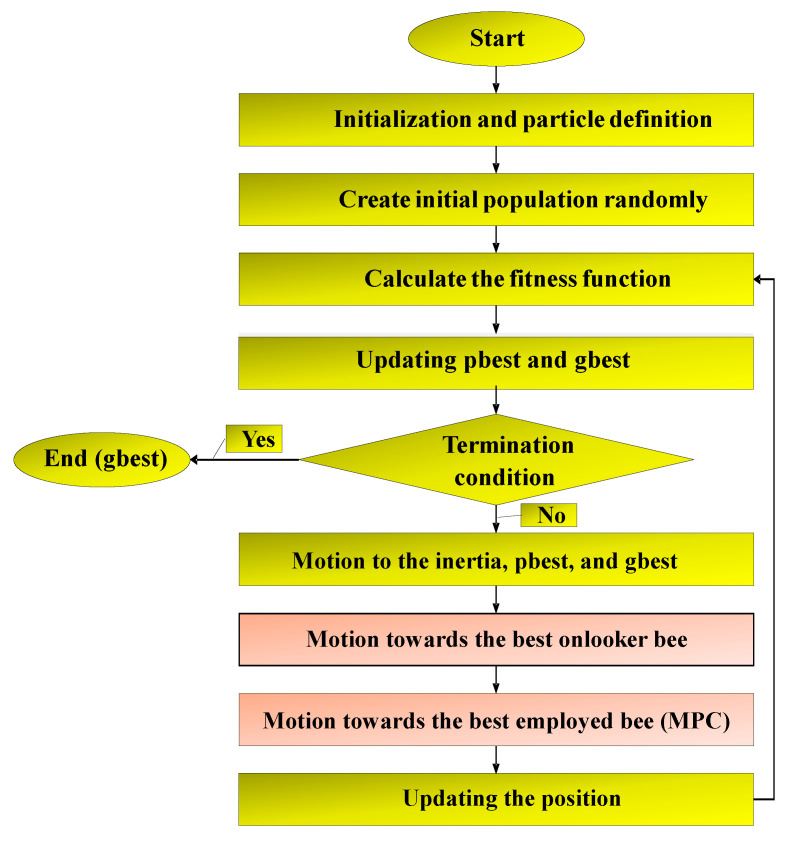
The flowchart of the proposed NSBPSO algorithm.

In standard PSO, the particle diversity gradually decreases as the particles move towards the personal best and global best. In this paper, due to the exploratory nature of the crossover operator, a multi-parent crossover is proposed to achieve highly varied solutions. In this operator, instead of using two employed bees, all employed bees participate in the crossover to create new solutions. When we use several best particles (as employed bees) to produce the new solutions, the obtained child bears less similarity to its parent, meaning that the solutions are diverse in the search space. Therefore, the multi-parent crossover operator improves the algorithm exploration. Figure 4 shows the example of the multi-parent crossover operator of the NSBPSO algorithm.

**Figure 5:**
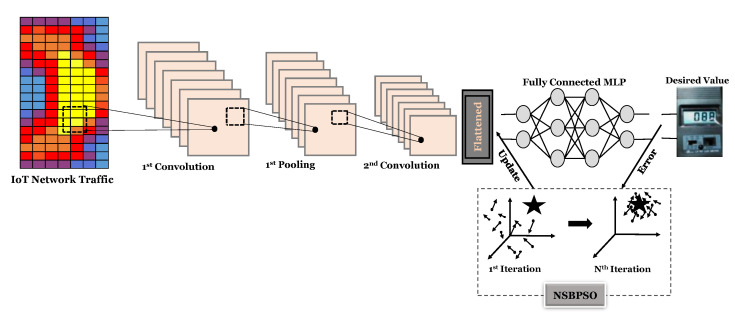
The overall schematic of the proposed model for detecting IoT network intrusions.

Therefore, Equation (1) is updated as follows and two new vectors are added to improve the PSO performance. Motion towards the best onlooker bee (from the neighborhood search operator) improves the algorithm’s exploitation. Motion to the best employed bee from the multi-parent crossover operator improves the algorithm’s exploration. Figure 5 shows the flowchart of the proposed NSBPSO algorithm.

(3) $$V_{id}\left(t+1\right)=\alpha V_{id}\left(t\right)+\beta \mathrm{rand}\left(0,\varphi_{1}\right)\left(P_{id}\left(t\right)-X_{id}\left(t\right)\right)+\beta \mathrm{rand}\left(0,\varphi_{2}\right)\left(P_{gd}\left(t\right)-\;X_{id}\left(t\right)\right)+\beta \mathrm{rand}\left(0,\varphi_{3}\right)\left(P_{od}\left(t\right)-X_{id}\left(t\right)\right)+\beta \mathrm{rand}\left(0,\varphi_{4}\right)\left(P_{ed}\left(t\right)-X_{id}\left(t\right)\right)$$

where $\beta \mathrm{rand}\left(0,\varphi_{3}\right)\;$ = a random number between zero and $\varphi_{3}$, $\beta \mathrm{rand}\left(0,\varphi_{4}\right)\;$ = a random number between zero and $\varphi_{4}$, $P_{od}\left(t\right)\;$ = the best onlooker bee from neighborhood search operator in dimension d, and $P_{ed}\left(t\right)\;$ = the best employed bee from the multi-parent crossover operator in dimension d. 

## 3. The Proposed IoT IDS Using the NSBPSO-Based Deep Architecture

In this section, we explain the proposed NIDS for the IoT, which mainly consists of the DCNN. The overall schematic of the proposed classifier is depicted in Figure 5. According to this figure, the input data passes through some convolution and pooling layers. After that, we use a fully connected MLP to classify the datasets. The fully connected MLP is trained by the proposed NSBPSO in order to achieve a higher classification and detection rate. More details will be discussed in the following subsections.

### 3.1. Datasets

We explain two network intrusion detection datasets named UNSW-NB15 [64] and Bot-IoT [65] in this section. 

#### 3.1.1. UNSW-NB15 Dataset

The raw network packets of the UNSW-NB15 dataset has been obtained from the IXIA Perfect-Storm tool in the Cyber Range Lab of the Australian Centre for Cyber Security in order to produce a hybrid of synthetic contemporary attack behaviors and real modern normal activities. UNSW-NB15 dominates the defects of the KDD99 dataset (for instance, no modern attacks, etc.) and has inchmeal become the most favorite dataset in the area of IoT intrusion detection in recent years. In the training dataset, the number of records is 175,341, whereas this number in the testing dataset decreases to 82,332. There are nine kinds of attacks in the UNSW-NB15 dataset named Fuzzers, Analysis, Backdoors, DoS, Exploits, Generic, Reconnaissance, Shellcode, and Worms.

#### 3.1.2. Bot-IoT Dataset

This is the latest IoT network intrusion detection dataset. The network environment in this dataset combined the normal and botnet traffic. In other words, Bot-IoT includes normal IoT network traffic as well as four different attacks named DoS, distributed DoS (DDoS), Reconnaissance, and Theft. Many IoT scenarios exist in Bot-IoT’s testbed, such as a weather station, a smart fridge, motion-activated lights, a remote-controlled garage door, and a smart thermostat. A huge number of traffic records exist in the raw CSV file of the Bot-IoT dataset, so we only use some parts of the traffic records for our simulations and experiments. In the training dataset, the number of terrific records is 364,562, whereas this number in the testing dataset decreases to 243,043. Table 1 shows more details of these datasets.

### 3.2. Training Deep Architecture Using the NSBPSO Algorithm

**Figure 6:**
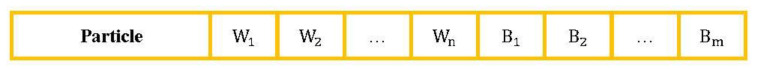
Particle definition in the NSBPSO algorithm.

In this paper, the NSBPSO algorithm is used to train deep learning, called the NSBPSO deep convolutional neural network (NSBPSO-DCNN). In the proposed algorithm, NSBPSO optimizes the weights and biases of the fully connected MLP in the DCNN. For NSBPSO modeling, one of the main tasks is to define a solution in the form of a particle. Figure 6 shows the definition of a particle in NSBPSO. The fitness function of proposed approach can be calculated as Equation (4).

(4) $$Mean\;Square\;Error\;\left(MSE\right)=\frac{1}{k}\;\overset{k}{\underset{i=1}{\sum }}{\left(O_{i}-D_{i}\right)}^{2}$$

where, k = the total number of samples, $O_{i}$ = system output, and $D_{i}$ = desire.

## 4. Simulation Results on the NID Datasets

In this section, the results of various hybrid deep architectures for intrusion detection in IoT systems are evaluated. The performance of the proposed NSBPSO algorithm is also evaluated in comparison with some widely-used and competitive metaheuristic algorithms, including the particle swarm optimization (PSO) algorithm, the artificial bee colony (ABC) algorithm, the iterated greedy algorithm (IG) [66], the improved crow search algorithm (I-CSA) [67], and the black widow optimization (BWO) algorithm [68]. All algorithms have been coded in MATLAB, and the calibration parameters of the algorithms have been shown in Table 2.

**Figure 7:**
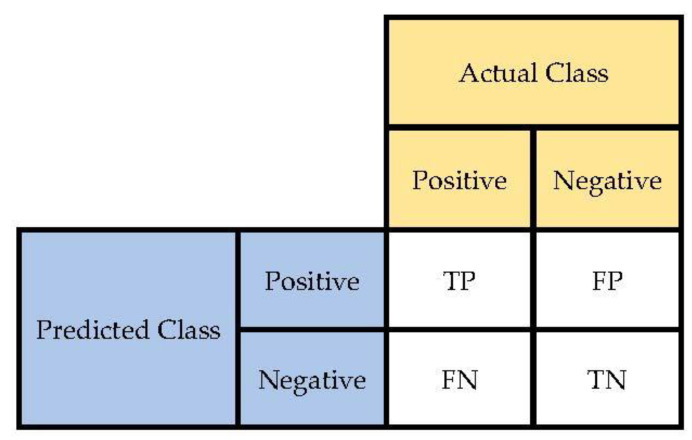
The confusion matrix.

For validation, sensitivity, accuracy, and specificity metrics are used to compare the performance of the deep architectures. These criteria are derived from the confusion matrix (as demonstrated in Figure 7) and can be calculated as Equations (5)–(7).

(5) $$Sensitivity=\frac{TP}{TP+FN}$$

(6) $$Specificity=\frac{TN}{TN+FP}$$

(7) $$Accuracy=\frac{TP+TN}{TP+FN+FP+TN}$$

where, TP = true positive, FN = false negative, TN = true negative, FP = false positive. Table 3 indicates the specificity, accuracy, and sensitivity of evolutionary deep learning models for intrusion detection in IoT systems. As can be seen, the NSBPSO-DCNN model indicates the highest ratios in accuracy, sensitivity, and specificity in training and testing datasets. NSBPSO-DCNN achieved 99.41% and 98.86% accuracy in the test and train datasets, respectively. NSBPSO-DCNN also achieved 99.86% and 99.03% sensitivity in the test and train datasets, respectively. 

**Figure 8:**
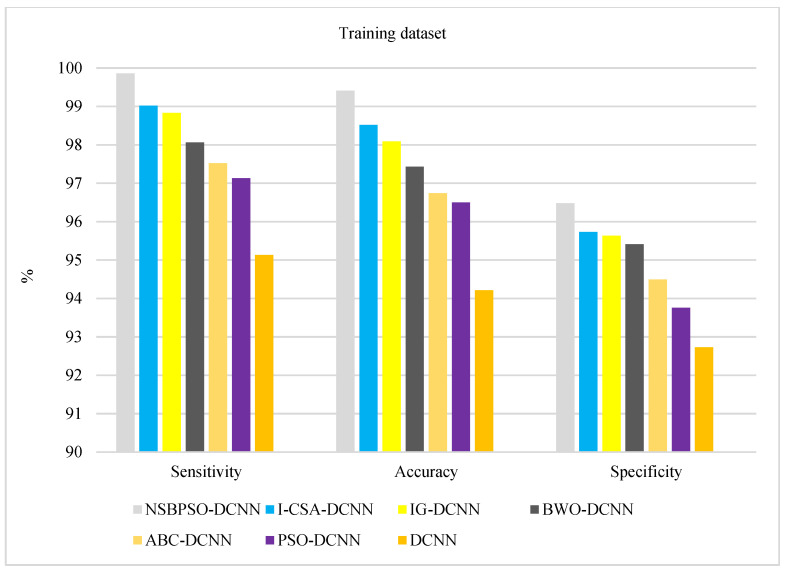
Comparison of the proposed architectures in the training dataset.

**Figure 9:**
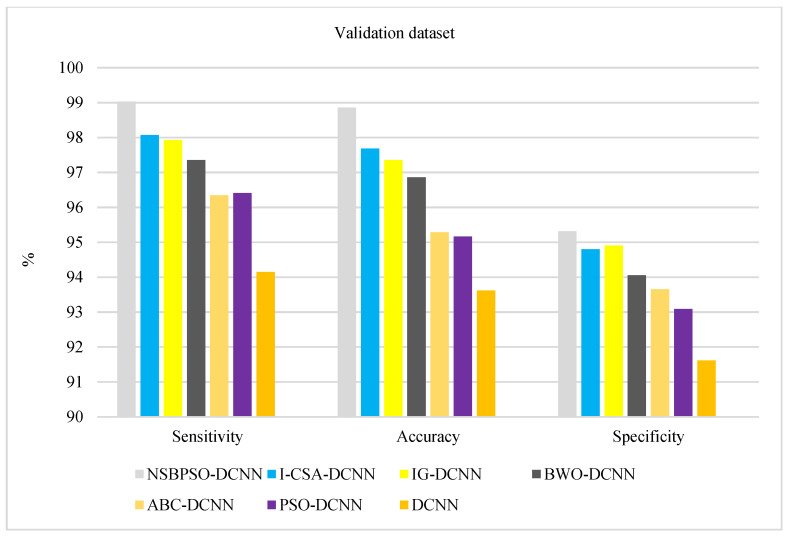
Comparison of the proposed architectures in the validation dataset.

Figure 8 and Figure 9 show the comparison of deep architectures in the training and validation datasets, respectively. According to Figure 8 and Figure 9, the rank of the architectures is: NSBPSO-DCNN, I-CSA-DCNN, IG -DCNN, BWO -DCNN, ABC-DCNN, PSO-DCNN, and Standard DCNN, respectively. The results of hybrid deep architectures in the test dataset show that the proposed architectures are well trained using meta-heuristic algorithms because the accuracy, specificity, and sensitivity of the different hybrid deep architectures in the test and train datasets are highly stable.

**Figure 10:**
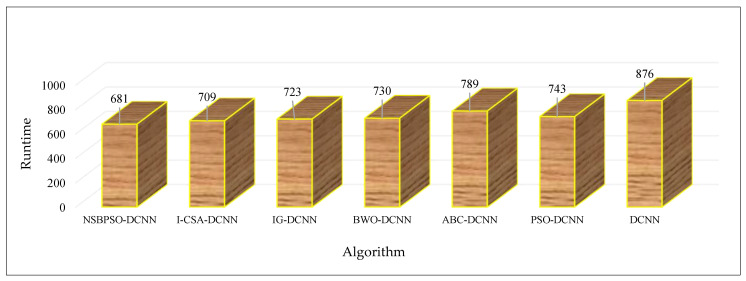
Comparison of the proposed algorithms.

Table 4 shows the trends of the accuracy and runtime of the proposed architectures in different epochs. According to this table, the NSBPSO-DCNN architecture has achieved the highest accuracy in the shortest runtime. The accuracy of the NSBPSO-DCNN, I-CSA-DCNN, IG-DCNN, BWO-DCNN, ABC-DCNN, PSO-DCNN, and DCNN architectures is 99.41%, 98.52%, 98.09%, 97.43%, 96.74%, 96.50%, and 94.21%, respectively. Figure 10 compares the total “Runtime” of the architectures. As can be seen, the runtime of NSBPSO-DCNN is less than other architectures. As mentioned in Section 2, to develop the proposed NSBPSO algorithm, employed bees and onlooker bees are used to improve the exploitation of the PSO algorithm. Multi-parent crossover is also proposed to improve the exploration of the algorithm. Hence, NSBPSO has provided the best results compared to other algorithms.

Table 5 indicates the value of the mean square error (MSE) for the proposed architectures. The proposed NSBPSO-DCNN model has a lower MSE than other methods. In the proposed NSBPSO, by considering several particles as the employed bees (global bests), different parts of the search space can be examined simultaneously. Therefore, it helps the algorithm to avoid being trapped in the local minimums. Therefore, the proposed NSBPSO-DCNN model has been useful for intrusion detection in IoT systems.

**Figure 11:**
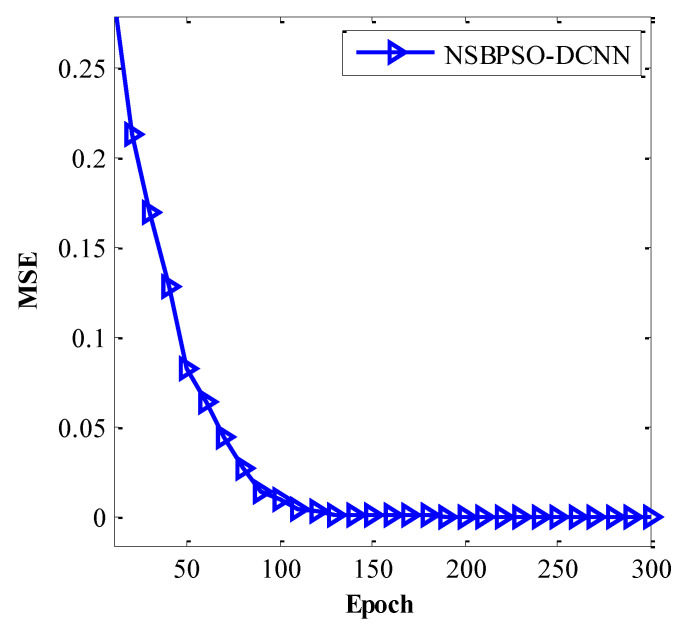
The convergence curve of the NSBPSO-DCNN architecture.

**Figure 12:**
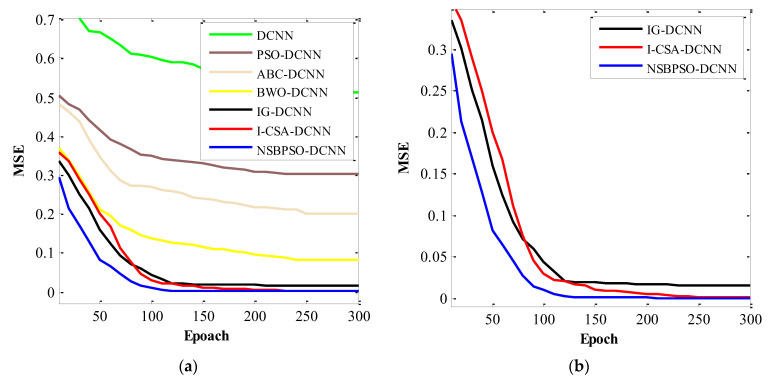
The convergence curve of the architectures: (**a**) All architecture; and (**b**) NSBPSO-DCNN, I-CSA-DCNN, IG-DCNN.

Figure 11 and Figure 12 show the convergence curve of the NSBPSO-DCNN and other architectures. The NSBPSO-DCNN architecture is close to its lowest MSE at epoch = 80. However, other architectures do not have good accuracy at epoch = 80. Subsequently, with an increasing epoch, NSBPSO-DCNN has achieved high stability and high convergence speed. As shown in Figure 12a, the convergence curve of the proposed NSBPSO-DCNN architecture is faster than the other architectures. The reason for NSBPSO’s superiority is the existence of two new operators. (a) The motion towards the best onlooker bee (from neighborhood search operator) improves the algorithm’s exploitation, and (b) the motion towards the best employed bee from the multi-parent crossover operator improves the algorithm’s exploration. Figure 12b shows the details of the convergence curves.

A nonparametric statistical test called Wilcoxon has been used to show the significant differences between all models. The Wilcoxon test is applied to measure the similarity of two dependent degree-scale samples. Derrac et al. [69] provided the full details of this nonparametric statistical test. All architectures have been implemented with 25 runs for intrusion detection in IoT systems. The mean values of the fitness function were normalized and then the Wilcoxon test results were obtained using SPSS software. Table 6 shows the R^+^, R^−^, and *p*-value for all NSBPSO-DCNN pairwise comparisons. As shown in Table 6, NSBPSO-DCNN shows an improvement versus I-CSA-DCNN, IG-DCNN, and BWO-DCNN with a level of significance *α* = 0.05, and versus ABC-DCNN, PSO-DCNN, and Standard DCNN with a level of significance *α* = 0.01. According to the results, NSBPSO-DCNN has a strong performance compared to the other algorithms.

## 5. Conclusions and Discussion

This paper developed a novel training algorithm for better tuning the parameters of the DCNN to accurately detect intrusion in IoT networks. Deep learning-based approaches could have improved the accuracy of the NIDS, though there were still some important features that needed to be improved, including achieving a higher detection rate and decreasing the computational cost. To do so, first, a novel modified PSO algorithm named the NSBPSO algorithm has been introduced to improve the exploitation and exploration abilities of the PSO algorithm. After that, we used the advantages of the NSBPSO algorithm to optimally train the deep architecture as our network intrusion detector in order to obtain better accuracy and performance. For evaluating the performance of the NSBPSO-based DCNN, we used two network intrusion detection datasets named UNSW-NB15 and Bot-IoT to evaluate the accuracy and performance of the proposed classifier. The experiment results have shown that the proposed NIDS has the best accuracy and performance in comparison with other state-of-the-art schemes.

## Figures and Tables

**Table 1:** Summary of the UNSW-NB15 [64] and Bot-IoT [65] datasets. Reprinted with permission from Ref. [52]. Copyright 2021 IEEE.

Dataset	Category	Training Dataset	Testing Dataset
UNSW-NB15	Normal	56,000	37,000
	Fuzzers	18,184	6062
	Analysis	2000	677
	Backdoors	1746	583
	DoS	12,264	4089
	Exploits	33,393	11,132
	Generic	40,000	18,871
	Recon.	10,491	3496
	Shell	1133	378
	Worms	130	44
	Total	175,341	82,332
Bot-IoT	Normal	286	191
	DoS	146,293	97,529
	DDos	163,287	108,858
	Recon.	54,649	36,433
	Theft	47	32
	Total	364,562	243,043

**Table 2:** The parameters settings of the algorithms.

Algorithm	Parameter	Value
NSBPSO	The inertial movement rate (α)	0.08
	The movement toward the best personal experience rate (Φ1)	0.56
	The movement toward the best global experience rate (Φ2)	0.84
	The movement toward the best onlooker bee from the neighborhood search rate (Φ3)	0.61
	The movement toward the best employed bee from the multi-parent crossover rate (Φ4)	0.59
	Population size	100
	Iteration	300
I-CSA	Flight length (fl)	2
	Awareness probability (AP)	0.1
	Population size	100
	Iteration	300
IG	T	0.4
	d	4
	Number of scout bees (population size)	100
	Iteration	300
BWO	Procreate rate (PP)	0.62
	Mutation rate (P_M_)	0.23
	Cannibalism rate (CR)	0.46
	Population size	100
	Iteration	300
ABC	Number of onlooker bees	90
	Number of employed bees	50
	Number of scout bees (population size)	100
	Iteration	300
PSO	The inertial movement rate (α)	0.11
	The movement toward the best personal experience rate (Φ1)	0.61
	The movement toward the best global experience rate (Φ2)	0.91
	Population size	100
	Iteration	300

**Table 3:** The results of the proposed algorithms for intrusion detection in IoT systems.

Deep Architectures	Training Dataset			Validation Dataset		
	Sensitivity	Specificity	Accuracy	Sensitivity	Specificity	Accuracy
NSBPSO-DCNN	0.9986	0.9648	0.9941	0.9903	0.9532	0.9886
I-CSA-DCNN	0.9902	0.9573	0.9852	0.9807	0.9480	0.9769
IG-DCNN	0.9883	0.9563	0.9809	0.9793	0.9491	0.9736
BWO-DCNN	0.9806	0.9541	0.9743	0.9736	0.9406	0.9686
ABC-DCNN	0.9752	0.9449	0.9674	0.9635	0.9366	0.9529
PSO-DCNN	0.9713	0.9376	0.9650	0.9641	0.9309	0.9517
Standard DCNN	0.9513	0.9273	0.9421	0.9415	0.9162	0.9362

**Table 4:** Accuracy and runtime of the models for different epochs.

Architectures	Metric	Epoch									
		30	60	90	120	150	180	210	240	270	300
NSBPSO-DCNN	Accuracy (%)	91.15	91.88	92.89	94.54	95.84	97.91	98.63	98.88	99.25	99.41
	Runtime (s)	74	145	196	275	321	384	462	521	598	681
I-CSA-DCNN	Accuracy (%)	90.16	90.89	91.76	93.60	94.79	95.50	96.98	97.95	98.21	98.52
	Runtime (s)	91	169	224	296	351	422	498	543	601	709
IG-DCNN	Accuracy (%)	89.19	90.47	91.85	92.19	93.59	94.90	96.48	97.43	97.89	98.09
	Runtime (s)	101	175	246	296	361	429	514	596	632	723
BWO-DCNN	Accuracy (%)	87.72	89.63	90.18	91.85	92.06	92.89	94.73	96.48	97.09	97.43
	Runtime (s)	110	185	239	310	389	435	520	599	649	730
ABC-DCNN	Accuracy (%)	89.18	90.19	91.08	91.73	92.76	93.09	94.19	94.81	95.12	96.74
	Runtime (s)	136	210	269	314	395	452	576	641	709	789
PSO-DCNN	Accuracy (%)	84.19	86.81	89.72	91.29	92.18	93.18	93.98	94.10	95.29	96.50
	Runtime (s)	115	196	267	32	406	459	534	612	693	743
DCNN	Accuracy (%)	78.85	83.49	86.79	89.12	90.13	90.83	91.45	92.71	93.28	94.21
	Runtime (s)	159	274	368	406	479	563	631	729	803	876

**Table 5:** The value of MSE for the proposed architectures.

Deep Learning Architectures	Mean Square Error (MSE)	
	Training Dataset	Validation Dataset
NSBPSO-DCNN	0.00010	0.00053
I-CSA-DCNN	0.00109	0.03012
IG-DCNN	0.01456	0.05106
BWO-DCNN	0.08186	0.10456
ABC-DCNN	0.20145	0.43296
PSO-DCNN	0.30156	0.58325
Standard DCNN	0.51256	0.74123

**Table 6:** The results of architectures in the nonparametric statistical test (Wilcoxon test).

Comparison of Algorithm	R^+^	R^−^	*p*-Value	Level of Significance (α)
NSBPSO-DCNN versus I-CSA-DCNN	33	22	0.074	α = 0.05
NSBPSO-DCNN versus IG-DCNN	35	20	0.053	α = 0.05
NSBPSO-DCNN versus BWO-DCNN	38	17	0.041	α = 0.05
NSBPSO-DCNN versus ABC-DCNN	43	12	0.007	α = 0.01
NSBPSO-DCNN versus PSO-DCNN	45	10	0.004	α = 0.01
NSBPSO-DCNN versus Standard DCNN	50	5	0.002	α = 0.01

## Data Availability

Not applicable.
